# Thyroid Storm Presenting as Acute Liver Failure in a Patient with Graves’ Disease

**DOI:** 10.7759/cureus.10333

**Published:** 2020-09-09

**Authors:** Muhammad Hashim Hayat, Zorays Moazzam, Ioannis A Ziogas, Amman Yousaf, Maham Hayat

**Affiliations:** 1 Internal Medicine: Gastroenterology, Vanderbilt University Medical Center, Nashville, USA; 2 Internal Medicine, Aga Khan University Hospital, Karachi, PAK; 3 Department of Surgery, Division of Hepatobiliary Surgery and Liver Transplantation, Vanderbilt University Medical Center, Nashville, USA; 4 Radiology, Hamad General Hospital, Doha, QAT; 5 Radiology, Services Institute of Medical Sciences, Lahore, PAK; 6 Department of Digestive Diseases and Nutrition, University of Oklahoma Health Sciences Center, Oklahoma, USA

**Keywords:** acute liver failure, thyrotoxicosis, hyperthyroidism

## Abstract

Although mild elevations in liver enzymes are relatively common in thyrotoxicosis, acute liver failure (ALF) is an infrequent occurrence. Despite the emergence of liver transplantation as a viable treatment for ALF, survival rates continue to remain unsatisfactory, coupled with higher rates of postoperative complications and re-transplantation in this group. We present the disease course, management, and outcome of a patient with Graves’ disease who presented with ALF secondary to thyroid storm. Thyrotoxicosis should always be included in the differential diagnosis of ALF, as timely diagnosis and management are crucial in achieving survival.

## Introduction

Thyroid storm is an acute exacerbation of pre-existing hyperthyroidism that can result in a life-threatening state secondary to multiorgan failure. Excess thyroid hormone results in the sympathetic nervous system's upregulation, with synergism being observed between thyroid hormones and catecholamines. Diagnosis is typically established based on clinical manifestations, including hyperthermia, tachycardia, congestive heart failure, atrial fibrillation, neurological symptoms, and gastrointestinal dysfunction. Stressful events, such as surgery, trauma, pregnancy, infections, are the most common thyroid storm triggers. A high mortality rate of 20%-30% warrants prompt therapy to manage thyroid storm [1]. The most frequent underlying pathology of hyperthyroidism is Graves' disease, with an annual incidence of 20-50 cases per 100,000 people [2]. Deranged liver function tests (LFTs) are not uncommon in patients with thyroid storm; however, acute liver failure (ALF) at presentation is infrequent. ALF is defined as the development of encephalopathy and coagulopathy in the absence of pre-existing liver disease and with an illness of fewer than twenty-six weeks in duration [3]. ALF can manifest as cerebral edema, cardiac arrhythmias, pulmonary edema, disseminated intravascular coagulation, acute renal failure, hypoglycemia, and impaired immune response. As liver transplantation has emerged as a viable treatment for ALF, survival rates have improved from 15% to 60%-80% [4]. In this article, we report a patient with a history of Graves' disease presenting with ALF secondary to thyroid storm, the disease course, management, and outcome of this rare occurrence.

## Case presentation

**Figure 1:**
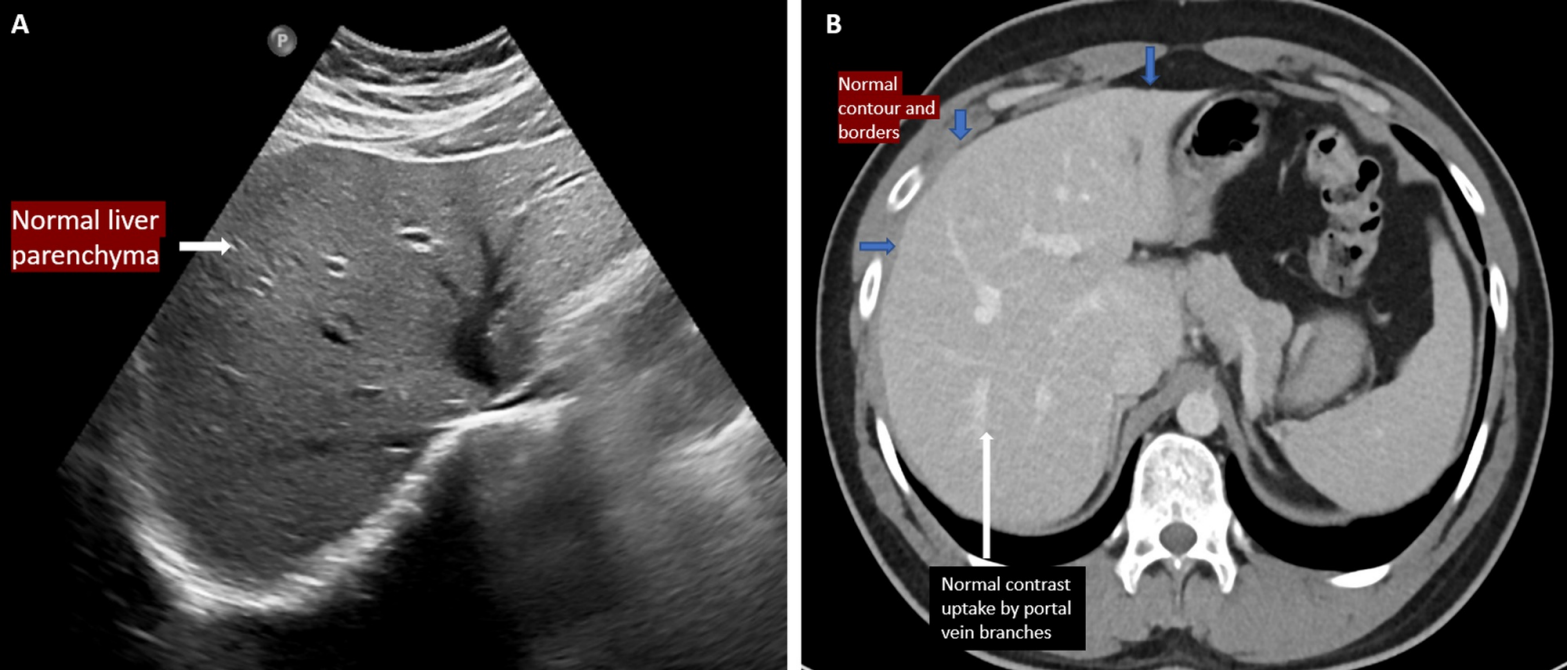
A selected image of a grey-scale ultrasound abdomen shows normal parenchymal echotexture, no focal lesion, or biliary dilatation (1A). A selected axial section of a contrast-enhanced CT scan of the abdomen demonstrates no focal lesion or collection in the liver or perihepatic fluid (1B).

A 22-year-old female patient with a medical history of Graves' disease (on propranolol), presented to our hospital in a markedly obtunded state with jaundice. She had a one-week history of abdominal pain, nausea, and vomiting. Here presenting vitals were; heart rate: 89 beats per minute, respiratory rate: 25 breaths per minute, blood pressure: 90/60 mmHg, and temperature 38.1 Celcius. Initially, the patient was resuscitated with fluids, and symptomatic treatment was provided. Laboratory workup on admission was significant for a total bilirubin of 17.3 mg/dL (direct bilirubin: 14.3 mg/dL), aspartate aminotransferase of 964 IU/L, alanine aminotransferase of 293 IU/L, alkaline phosphatase of 146 IU/L, INR of 3.5, and ammonia of 200 μg/dL. Ultrasound and computed tomography scan of the abdomen revealed no focal liver abnormality, and no intra- or extrahepatic biliary dilatation (Figure *1*).

A detailed history from the family revealed no history of using any hepatotoxic medications, herbal supplements, or alcohol abuse by the patient. Other causes of ALF, including viral hepatitis and autoimmune hepatitis, were ruled out. Further workup revealed a thyroid-stimulating hormone of <0.008 μIU/L, free T3 of 17.2 pg/mL, and free T4 of 5.6 ng/dL. Based on these findings, the patient was managed for thyroid storm with steroids, propylthiouracil (PTU), and methimazole. As the patient's thyroid function improved with treatment, the patient's conscious level improved, the LFTs started to trend down and normalized at a one-month follow-up visit. The patient was counseled about compliance with her medications and a regular follow-up with the endocrinology clinic.

## Discussion

The existing literature on ALF secondary to thyroid storm is limited to a few case reports. Almost 15% to 76% of patients with untreated hyperthyroidism present with mildly elevated LFTs, ALF in this group of patients is extremely uncommon [1,5-9]. The exact mechanism of ALF in thyroid storm is still controversial. Increased T3 directly causing mitochondria-mediated hepatocyte apoptosis, hepatic ischemia secondary to peripheral vasodilation, and excess thyroid hormone causing right-sided heart failure, leading to increased backpressure and hepatic failure, are the possible mechanisms of ALF in thyrotoxicosis [10]. The pattern of hepatic injury can range from mild derangement of LFTs to liver failure. Our patient met the criteria for definite thyroid storm, with central nervous system dysfunction (obtunded state), gastrointestinal dysfunction (nausea, vomiting), and hepatic dysfunction (jaundice, total bilirubin > 3 mg/dL) [11]. A potential obstacle to the resolution of this condition is the nature of the therapy that is available. Although PTU is considered first-line treatment for thyroid storm, a known side-effect of this medication is ALF via a proposed hypersensitivity reaction. It has been suggested that PTU is the second most common drug following acetaminophen to cause ALF requiring liver transplantation [12]. Moreover, methimazole has been rarely associated with hepatotoxicity two to twelve weeks after the onset of therapy. Nevertheless, in our patient, a thorough history ruled out any hepatotoxic medications use, including PTU, and laboratory tests suggested that the ALF occurred due to thyroid storm. Furthermore, symptoms improved after the administration of steroids, PTU, and methimazole.

In our case, medical management of thyroid storm resulted in the down-trending of LFTs. However, in case of a rapid deterioration of liver function tests despite supportive treatment, emergent liver transplantation remains the only definite treatment. However, patients with ALF tend to have significantly worse outcomes after liver transplantation than the general population, with higher rates of immediate postoperative complications, re-transplantation, and a longer duration of stay in the intensive care unit [13]. As a result, prompt diagnosis and medical treatment of ALF is essential, with an urgent transition to surgery if the patient’s condition continues to deteriorate.

## Conclusions

This article highlights the rare association of thyrotoxicosis and ALF. It also demonstrates the diagnostic and management challenges encountered in ALF in the thyroid storm setting. Furthermore, we can conclude that early diagnosis and prompt medical treatment of concomitant thyrotoxicosis and ALF can prevent ALF's detrimental consequences and the need for a liver transplant. We also suggest that physicians must keep thyrotoxicosis in the differential diagnosis of ALF causes, especially in patients with a known history of thyroid dysfunction.
